# Tumor Necrosis Factor Receptor-Associated Protein 1 Protects against Mitochondrial Injury by Preventing High Glucose-Induced mPTP Opening in Diabetes

**DOI:** 10.1155/2020/6431517

**Published:** 2020-03-06

**Authors:** Lerong Liu, Lingxiao Zhang, Jiangpei Zhao, Xiangyu Guo, Yuanyuan Luo, Wenli Hu, Tongfeng Zhao

**Affiliations:** ^1^Department of Endocrinology, The Six Affiliated Hospital of Sun Yat-sen University, Guangzhou, Guangdong, China; ^2^Department of Neurology, The Six Affiliated Hospital of Sun Yat-sen University, Guangzhou, Guangdong, China; ^3^Guangdong-Hongkong-Macau Institute of CNS Regeneration, Ministry of Education CNS Regeneration Collaborative Joint Laboratory, Jinan University, Guangzhou, Guangdong, China; ^4^Department of Critical Care Medicine, The Six Affiliated Hospital of Sun Yat-sen University, Guangzhou, Guangdong, China

## Abstract

Diabetic kidney disease (DKD) has become the leading cause of end-stage renal disease worldwide. Renal tubular epithelial cell apoptosis and tubular atrophy have been recognized as indicators of the severity and progression of DKD, while the mechanism remains elusive. Tumor necrosis factor receptor-associated protein 1 (TRAP1) plays critical roles in apoptosis. The aim of this study was to investigate the protective role TRAP1 plays in DKD and to study the potential underlying mechanisms. TRAP1 expression was decreased, and mitochondria were injured in NRK-52e cells under high-glucose (HG) conditions. The overexpression of TRAP1 ameliorated HG-induced apoptosis, increased cell viability, maintained mitochondrial morphology, adenosine triphosphate (ATP) levels, and mitochondrial membrane potential (MMP), and buffered oxidative stress, whereas TRAP1 knockdown aggravated these effects. The protective effects of TRAP1 may be exerted via the inhibition of mitochondrial permeability transition pore (mPTP) opening, and the damage caused by TRAP1 knockdown can be partially reversed by treatment with the mPTP opening inhibitor cyclosporin A (CsA). In vivo, TRAP1 expression upregulation by AAV2/9 injection prevented renal dysfunction, ameliorated histopathological changes, maintained mitochondrial morphology and function, and reduced apoptosis and reactive oxygen species (ROS) in STZ-treated DKD rats. Thus, our results suggest that TRAP1 ameliorates diabetes-induced renal injury by preventing abnormal mPTP opening and maintaining mitochondrial structure and function, which may be treated as a potential target for DKD treatment.

## 1. Introduction

Diabetic kidney disease (DKD), one of the most frequent complications of both type 1 and 2 diabetes mellitus, has become the major cause leading to chronic kidney disease and end-stage renal disease (ESRD) worldwide [1, 2]. The proximal tubule is thought to be a key player in the pathology of DKD [3]. The apoptosis, rather than necrosis, of tubular epithelial cells may predominantly account for lingering tubular atrophy, which has been recognized as an indicator of renal disease severity and progression [4, 5].

Mitochondria, the “cell's power plants,” are highly dynamic organelles that meet the metabolic demands of cells. Mitochondria are involved in crucial apoptosis pathways [6]. High-glucose (HG) stimulators prompt mitochondrial dysfunction, along with damaged energy homeostasis and increased intracellular concentrations of reactive oxygen species (ROS), which are reported to eventually lead to cellular dysfunction or death [2, 7]. The kidney is a mitochondrially rich and highly metabolic organ that requires a large number of mitochondria to function normally. Hence, protection from mitochondrial dysfunction plays a critical role in ameliorating the apoptosis of renal tubular cells and thus may be a feasible strategy for the treatment of DKD.

Tumor necrosis factor receptor-associated protein 1 (TRAP1), a member of the heat-shock protein 90 (HSP90) family, is abundantly expressed in the kidney and primarily localized in the mitochondrial matrix [8, 9]. The function of TRAP1 in mitochondria is highly complex and controversial. In nontumor cells, TRAP1 plays a regulatory role in stress sensing in mitochondria for cellular adaption to environmental injury in different disease models [10–12]. In tumor cells, TRAP1 may function as an oncogene or tumor suppressor in different types of cancer [13–16]. TRAP1 is involved in various kinds of cellular processes, but thus far, little has been reported about the role of TRAP1 in DKD.

In this study, the metabolic-regulatory role of TRAP1 and the underlying mechanisms in DKD were explored in tubular epithelial cell lines and adeno-associated virus (AAV) 2/9-infected diabetic rats. We detected its role in mitochondrial injury, oxidative stress, and apoptosis induced by hyperglycemia to provide new perspectives on tubular injury and provide new therapeutic strategies for DKD treatment.

## 2. Methods

### 2.1. Cell Culture

Normal rat kidney proximal tubular cells (NRK-52E; negative for mycoplasma contamination) were obtained from the Chinese Academy of Sciences (Shanghai, China) and cultured in Dulbecco's modified Eagle's medium (DMEM, Gibco Life Technologies, Waltham, MA, USA) supplemented with 5% fetal bovine serum (FBS) and 1% antibiotics (vol./vol.) at 37°C with 5% CO_2_ in a humidified incubator. Cells used for experiments were harvested during logarithmic growth phase.

### 2.2. Animals

All animals received humane care according to the National Institutes of Health (USA) guidelines. Sprague-Dawley rats were originally obtained from the Animal Center of Sun Yat-sen University (Guangzhou, China). Male rats aged 8 to 10 week old were used for experiments. Before the studies, the animals were acclimatized for at least 2 weeks. To induce diabetes, following 12 h of fasting, experimental model rats were treated with a single intraperitoneal injection of 50 mg/kg streptozotocin (STZ; Sigma-Aldrich, St. Louis, MO, USA) dissolved in citrate buffer (pH 4.5); the control rats received citrate buffer alone and served as the normal control group. Additional anesthesia and analgesia were given based on the response to a tail pinch. Diabetic rats were defined as having a random blood glucose level greater than 16.67 mmol/l after STZ injection. The expression of TRAP1 was driven by the CMV promoter, in fusion with the Flag tag at the C-terminus. AAV vectors were serotyped with AAV2/9 coat protein and packaged by Obio Technology Co. (Shanghai, China). Viral titers were approximately 2 × 10^13^ genomic copies per ml for pAAV-CMV-Trap1-P2A-EGFP-3FLAG-CW3SL and 5 × 10^13^ genomic copies per ml for the empty vector pAAV-CMV-MCS-EGFP-3FLAG-CW3SL. The number of injected viral particles of pAAV-CMV-Trap1-P2A-EGFP-3FLAG-CW3SL and the empty vector control was 8 × 10^11^. Rats were randomly divided into four groups as follows:

*Group 1*: control group, normal rats received citrate buffer on the day of injection

*Group 2*: diabetes group, rats received an injection of STZ

*Group 3*: diabetes group + empty viral vector, rats received a tail-vein injection of 0.1 ml of empty viral vector after STZ injection

*Group 4*: diabetes group + TRAP1 AAV-2/9, rats received a tail-vein injection of an equal volume of 0.1 ml of TRAP1 AAV2/9 after STZ injection

After blood glucose levels stabilized for 5 days, AAV was injected into rats of group 3 and 4 via the tail vein. DKD developed 3 months after STZ injection. At the end of the experiment, blood and urine were collected. Rat kidneys were rapidly dissected and weighed for subsequent analyses.

### 2.3. Biochemical Index Analysis

In rat models, 24 h urine samples were collected at 48 h before euthanization. The body weight and kidney weight were recorded. The renal index (RI) is the ratio of kidney weight (KW) to body weight (BW) and was calculated by the formula RI (KW/BW) = [left kidney weight (mg) + right kidney weight (mg)]/(2∗body weight)(g) [17]. Urine, blood, and kidney samples were collected for subsequent analyses. The levels of total cholesterol (TC), total triglyceride (TG), high-density lipoprotein (HDL), low-density lipoprotein (LDL), blood glucose, fructosamine (FRU), urea, and total albumin in serum and urine and the creatinine clearance rate (Ccr) were detected using commercially available kits (Roche, USA). All kits were used according to the manufacturer's instructions.

### 2.4. Histological Analysis

Separated kidney cortexes were fixed with 4% paraformaldehyde for paraffin embedding and histopathological analysis or 4% precooled glutaraldehyde for transmission electron microscopy (TEM). The remaining kidney cortex tissues were stored in liquid nitrogen for subsequent experiments. The fixed kidney tissues were sectioned at 3 *μ*m thickness and stained with hematoxylin and eosin (H&E), periodic acid-Schiff (PAS), Sirius red, and Masson's trichrome stain using commercial kits (Servicebio, Wuhan, China) according to the manufacturer's instructions. The slides were examined by electron light microscopy (400^∗^ amplification; Olympus, Japan).

### 2.5. ATP Measurement

In vitro, ATP was measured using the CellTiter-Glo Luminescent Cell Viability Assay kit (Promega, USA). After samples were centrifuged to remove cell debris, the supernatant was added to the substrate solution. For the renal cortex, the ATP content was evaluated by using an enhanced ATP assay kit (Beyotime, Wuhan, China) according to the manufacturer's instructions. A total of 20 mg of renal cortex tissue was added to 0.1 ml of lysis buffer, and the supernatant was used to detect the ATP content. The tests were conducted in 96-well plates with opaque walls with clear bottoms. The luminescence was recorded using a luminometer (Thermo Fisher Scientific, MA, USA). All data were normalized to the cell number and protein concentration.

### 2.6. Mitochondrial Membrane Potential (MMP) Measurement

The JC-1 probe (Beyotime Institute of Biotechnology, Shanghai, China) and tetramethylrhodamine methyl ester staining (Thermo Fisher Scientific, MA, USA) were used to evaluate MMP. Cells were incubated with PBS or HBSS containing JC-1 or tetramethylrhodamine methyl ester for 30 min at 37°C, washed twice with PBS, and subjected to fluorescence analysis with a fluorescence microscope or by flow cytometry.

### 2.7. Western Blot and qRT-PCR Analysis

Total protein from cells and kidney cortex homogenates were collected and lysed in RIPA buffer (Beyotime Institute of Biotechnology, Shanghai, China). The protein concentration of extracts was determined by BCA assay (Thermo Fisher Scientific, USA). Equal amounts of protein were separated by 8% SDS-PAGE and transferred to moist polyvinylidene fluoride membranes (Millipore Corp, USA). After being blocked in 5% BSA for 1 h, the membranes were probed overnight at 4°C with antibodies against TRAP1 (1 : 1000; Novus Biologicals, USA) and GAPDH (1 : 5000; Proteintech, USA). The membranes were washed and incubated with an HRP-conjugated antibody (1 : 5000; MultiScience, China) in blocking buffer for 120 min. Immunoreactive bands were visualized with ECL reagent (Millipore, MA, USA) using a ChemiDoc Imaging System (Bio-Rad, CA, USA).

Total RNA samples were extracted using TRIzol reagent (Takara, Otsu, Japan) and reverse transcribed to cDNA using Prime-ScriptTM RT reagent kits with gDNA eraser (Takara, Otsu, Japan). The mRNA expression levels were measured using real-time PCR on Applied Biosystems 7500 (Carlsbad, USA). GAPDH expression was used as a control for normalization, and the dynamic variations were calculated according to the 2^–*ΔΔ*Ct^ relative quantification method. Full-length rat TRAP1 cDNA was amplified by PCR using the following primers: TRAP1-forward: 5′-CAGGTGCTGCCAGAAATGGA-3′, TRAP1-reverse: 5′-GATCTGGCGATTGTGCCAAG-3′; GAPDH- forward: 5′-ATTCAACGGCACAGTCAAGG-3′, GAPDH-reverse: 5′-CACCAGTGGATGCAGGGAT-3′.

### 2.8. Generation of TRAP1-Overexpressing or TRAP1-Silenced NRK-52e Cells

Lentiviral vectors expressing TRAP1 and lentiviral vectors expressing TRAP1 shRNA-1 (5′-GCTGACAAAGTTGAAGTCTAT-3′), shRNA-2 (5′-ACGAGGAATTCTACCGTTA-3′), and shRNA-3 (5′-AGAGCTAATGGCATGGATGAGAAATGCAC-3′; GeneCopoeia, Rockville, MD, USA) were amplified. The cells were transfected with Lenti-TRAP1 or Lenti-shRNA, selected using puromycin for 10 d and verified by the measurement of mRNA and protein levels.

### 2.9. Apoptosis

The morphology of apoptotic cells was observed by nuclear staining with Hoechst 33258. Cells were stained with 10 mg/l Hoechst 33258 (Beyotime, Wuhan, China) at 37°C for 20 min. Cell images were obtained using a fluorescence microscope (Olympus IX73, Tokyo, Japan). Apoptosis of the treated cells was also determined by flow cytometry using the Annexin V-APC/7-AAD Apoptosis kit (MultiSciences, Huangzhou, China). After the incubation period, cells were trypsinized without EDTA and collected and suspended in a binding buffer according to the manufacturer's instructions. The rate of apoptosis was evaluated using a flow cytometer (BD FACSCanto II, BD Biosciences, NY, USA). A caspase Glo assay (Promega, Madison, WI, USA) was performed following the manufacturer's instructions. Cells were seeded in a black 96-well plate and treated, and 100 *μ*l of caspase Glo substrate was added per well. The plate was incubated for 90 min in the dark and read on a microplate reader (Thermo Fisher Scientific, MA, USA).

### 2.10. Cell Viability Assay

Cell viability was estimated using a CCK-8 assay according to the manufacturer's instructions (Dojindo Molecular Technologies, Kumamoto, Japan). Briefly, CCK-8 was added to each well at 0, 24, and 48 h after culturing and then the plate was incubated for 3 h at 37°C and measurements were taken. The absorbance at 450 nm was detected using a microplate reader (Thermo Fisher Scientific, MA, USA).

### 2.11. TEM

The changes in mitochondrial ultrastructure were confirmed by TEM (Hitachi HT7700, TO, Japan). Renal cortexes and NRK-52e cells were fixed in 4% glutaraldehyde solution at 4°C. Specimens were cut into sections, stained with uranyl acetate and leas citrate, and subjected to observation.

### 2.12. Measurement of Intracellular Reactive Oxygen Species (ROS) and Mitochondrial Superoxide Generation

Cells were incubated with CellROX® Deep Red Flow Cytometry Assay kit (Molecular Probes, MA, USA) or MitoSOX Red Assay (Molecular Probes, Eugene, OR, USA) in a medium for 30 min at 37°C in the dark. The ROS levels of tissues were detected on frozen slides by fluorescent probe using a commercial ROS Red kit (G1045, Servicebio, Wuhan, China). Subsequently, the fluorescence were examined using a flow cytometer (BD FACS Canto II, BD Bioscience, NY, USA) or a laser scanning confocal microscopy (Leica TCS SP8, WE, Germany). Total and mitochondria-derived ROS levels were quantified by analyzing the fluorescence intensity with Image-Pro Plus software (Media Cybernetics, USA).

### 2.13. Evaluation of mPTP Opening

The mPTP opening was assessed with a MitoProbe™ Transition Pore Assay kit for flow cytometry (Molecular Probes, MA, USA). Cells were loaded with calcein AM, which diffused into the cells passively and accumulated in mitochondria and the cytosol to liberate the highly polar fluorescent dye calcein. The cytosolic fluorescence can be quenched by CoCl_2_ (cobalt chloride), while mitochondrial fluorescence is maintained. Opening the mPTP instigated the release of calcein AM from the mitochondria into the cytosol, which resulted in a reduction in fluorescence. The change of fluorescence was examined using a flow cytometer (BD FACS Canto II, BD Bioscience, NY, USA).

### 2.14. TUNEL Assay

Apoptosis was examined by a Terminal deoxynucleotidyl transferase-mediated dUTP nick-end labeling (TUNEL) staining kit (Thermo Fisher Scientific, MA, USA) according to the manufacturer's instructions. Staining was detected by confocal microscopy (Leica TCS SP8, WE, Germany).

### 2.15. Statistical Analysis

Data are presented as the mean ± SEM. Statistical analysis of the results was performed with SPSS 20.0 software (IBM Corporation, IL, USA). Multiple comparisons were made using one-way ANOVA and Dunnett's tests. Differences with a *p* value less than 0.05 were considered statistically significant.

## 3. Results

### 3.1. Effect of High Glucose on TRAP1 Expression and Mitochondria in NRK-52e Cells

**Figure 1:**
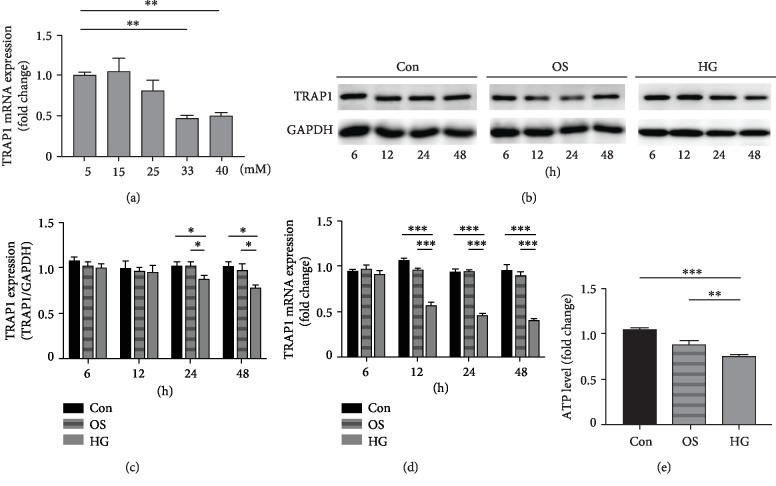
Effects of high glucose on TRAP1 levels and mitochondrial function in NRK-52e cells. (a) The levels of TRAP1 mRNA in NRK-52e cells (normalized to GAPDH) incubated in different glucose concentrations. (b, c) Cells were divided into three groups: normal glucose control group (Con, 5.5 mmol/l), osmotic control group (OS, 5.5 mmol/l glucose plus 27.5 mmol/l mannitol) and high-glucose group (HG, 33 mmol/l glucose). Representative western blot graphs and statistical analysis of TRAP1 and GAPDH at various incubation times. GAPDH was used as a protein loading control. (d) TRAP1 mRNA expression (normalized to GAPDH) was detected at different times. (e) ATP depletion was measured following high-glucose treatment for 48 h. ATP: adenosine triphosphate; GAPDH: glyceraldehyde 3-phosphate dehydrogenase. The results are presented as the mean ± SEM; *n* = 3‐5, ^∗^*p* < 0.05, ^∗∗^*p* < 0.01, and ^∗∗∗^*p* < 0.001 for each pair of groups indicated.

We first investigated the effect of various glucose concentrations on TRAP1 expression under HG conditions. The expression of TRAP1 mRNA was decreased by HG stimulation in NRK-52e cells in a dose-dependent manner at 48 h (Figure 1(a)). HG concentrations of 33 and 40 mM significantly decreased TRAP1 mRNA expression. Thus, 33 mM was the first glucose concentration inducing a significant downregulation of TRAP1 expression and was used as the HG stimulation condition in our study. To investigate the expression change of TRAP1 under HG conditions for different periods, we examined the levels of TRAP1 in NRK-52e cells. Mannitol served as an osmotic control for HG. TRAP1 protein levels decreased in HG condition at 24 h and 48 h (Figures 1(b) and 1(c)). Additionally, HG treatment yielded reduced TRPA1 mRNA expression after 12 h (Figure 1(d)). To examine the toxicity effects of HG on mitochondria, NRK-52e cells were exposed to HG for 48 h. Mitochondrial function was analyzed using ATP quantification and mitochondrial membrane potential (MMP). The level of ATP in HG stimulation conditions was significantly lower than that in highly osmotic conditions (Figure 1(e)). MMP loss was observed in the HG group with an increase in green fluorescence and a decrease in red fluorescence (ESM Fig. [Supplementary-material supplementary-material-1]). These results show that HG, more than high osmolarity, induced mitochondrial injury in NRK-52e cells, which may be associated with the decrease in TRAP1 expression.

### 3.2. TRAP1 Inhibited HG-Induced Apoptosis in NRK-52e Cells

**Figure 2:**
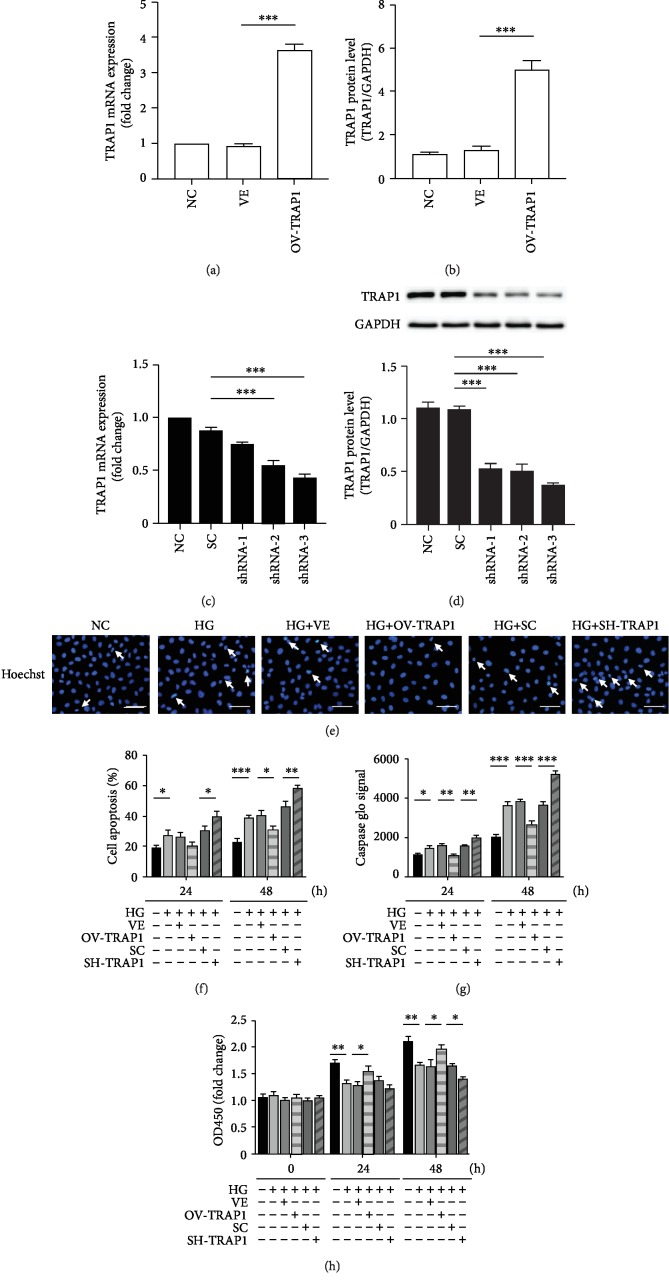
Effects of TRAP1 on apoptosis and cell viability following exposure of NRK-52e cells to HG. (a, b) Relative TRAP1 mRNA levels and protein levels were determined by RT-PCR and western blot following transfection with the OV-TRAP1 lentiviral vector. (c, d) TRAP1 mRNA levels and protein levels were determined following transfection with lentiviral vectors expressing shRNA-1, shRNA-2, and shRNA-3. (e) Cells were stained with Hoechst 33258 (blue), and arrows indicate apoptotic cells. (f) The apoptosis rates were detected by flow cytometry after APC/7AAD dual staining. (g) Graph depicting the fold increase in apoptosis was assessed by caspase Glo assay. (h) Cell viability was determined with a CCK-8 assay. The results are presented as the mean ± SEM; *n* = 3, ^∗^*p* < 0.05, ^∗∗^*p* < 0.01, ^∗∗∗^*p* < 0.001. VE: vector group; SC: scramble group.

To identify the effects of TRAP1 on HG-induced injuries, we performed TRAP1 overexpression and knockdown experiments by lentiviral infection in NRK-52e cells. The levels of TRAP1 mRNA and protein were significantly higher in cells infected with Lenti-TRAP1 than cells infected with the empty vector (VE) and negative control (NC; Figures 2(a) and 2(b)). Furthermore, PCR and western blot demonstrated that the greatest gene knockdown effect occurred in the shRNA-3 group, which was defined as the SH-TRAP1 group (Figures 2(c) and 2(d)). The transfection of cells with lentivirus significantly increased or reduced TRAP1 expression. Apoptosis and cell viability were detected to evaluate the cell survival capacity. Fluorescence microscopy with Hoechst 33258 staining and flow cytometric analyses using APC/7AAD staining and caspase-3 activity were performed to investigate apoptosis. At 48 h of HG treatment, TRAP1 overexpression decreased apoptosis, and TRAP1 silencing significantly increased apoptosis (Figure 2(e)). Similar results were obtained by flow cytometry after 24 and 48 h of HG treatment (Figure 2(f)). Representative images of apoptosis staining by APC/7AAD are shown in the supplementary material (ESM Fig. [Supplementary-material supplementary-material-1]). Caspase-3 activity is essential for regulating apoptosis as the executive protein. HG treatment enhanced caspase-3 activity in NRK-52e cells. The overexpression of TRAP1 led to a significant decrease in caspase-3 activity, whereas silencing of TRAP1 elicited a notable increase in caspase-3 activity at both 24 and 48 h (Figure 2(g)). Additionally, the effects of TRAP1 on cell viability were detected with the CCK-8 assay. TRAP1 expression upregulation enhanced NRK-52e cell viability at 24 h and 48 h. In contrast, the viability of TRAP1-knockdown cells was decreased at 48 h (Figure 2(h)). These results indicate that TRAP1 exerts an antiapoptotic effect on NRK-52e cells exposed to HG conditions.

### 3.3. TRAP1 Blocked HG-Induced Mitochondrial Damage in NRK-52e Cells

**Figure 3:**
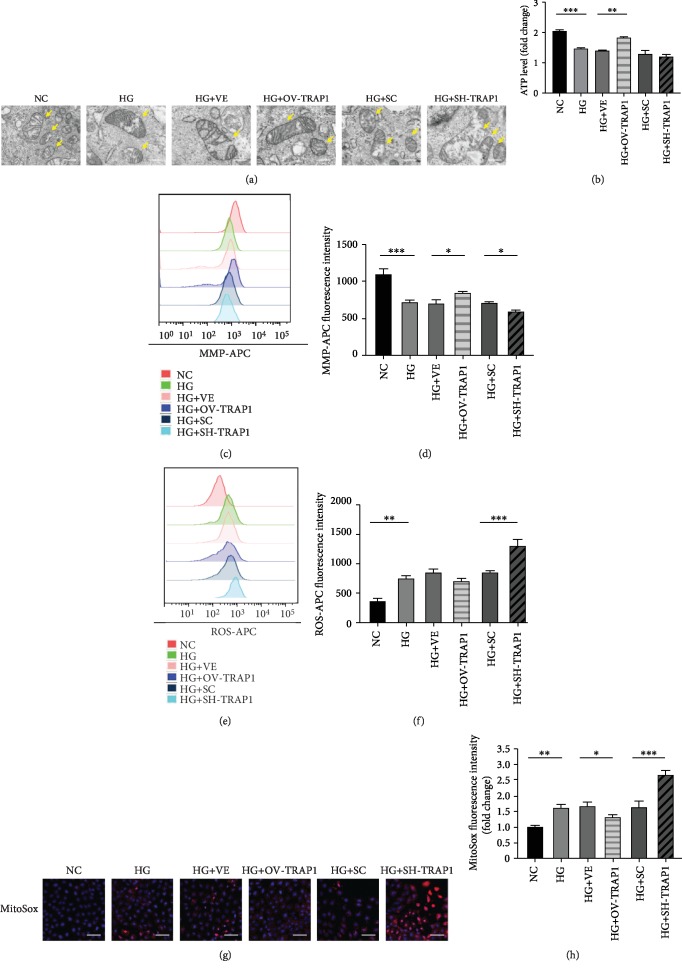
Effects of TRAP1 on mitochondrial morphology and dysfunction in NRK-52e cells after high-glucose injury. (a) Mitochondrial morphology was observed by TEM. Arrows indicate mitochondria; scale bar, 500 nm. (b) ATP depletion was measured using firefly luciferase. (c, d) Representative plots of MMP and statistical analyses were determined by flow cytometric analysis of tetramethylrhodamine ethyl ester-labeled NRK-52e cells. (e, f) Representative plots and statistical analysis of intracellular ROS in cells labeled with the fluorescent probe CellROX Deep Red and analyzed by flow cytometry. (g, h) Typical fluorescence photomicrograph and quantitative analysis of mitochondrial superoxide (red: MitoSox; blue: DAPI); scale bar, 50 *μ*m. ROS: reactive oxygen species. The results are presented as the mean ± SEM; *n* = 3, ^∗^*p* < 0.05, ^∗∗^*p* < 0.01, and ^∗∗∗^*p* < 0.001 for each pair of groups indicated.

To further investigate the mitochondrial morphology changes induced by HG, TEM was used to measure mitochondrial ultrastructural damage. In cells treated with HG for 48 h, the mitochondria showed severe damage with fracturing, swelling, and vacuolar structures. When TRAP1 expression was upregulated, the mitochondrial morphology displayed varying degrees of recovery, including few swollen and slightly broken organelles in TEM images. However, TRAP1 silencing aggravated the HG-induced alteration of the mitochondrial structure (Figure 3(a)). For mitochondrial function, the ATP content was higher in TRAP1-overexpressing cells than control vector-expressing cells at 48 h (Figure 3(b)). To further verify the TRAP1-mediated blockade of MMP loss in cells subjected to HG, we also evaluated cells using the tetramethylrhodamine methyl ester assay at 48 h of incubation. MMP was reduced under HG conditions, similar to the results detected by JC-1 above. The MMP reduction was inhibited by TRAP1 overexpression. The silencing of TRAP1 resulted in increased depolarization of MMP, which indicated increased mitochondrial ultrastructural damage (Figures 3(c) and 3(d)). Additionally, excessive ROS could cause oxidative stress and apoptosis. In NRK-52e cells, hyperglycemia increased cellular and mitochondrial ROS production. The overexpression of TRAP1 reduced mitochondrial ROS signals, and knockdown of TRAP1 exacerbated the production of total ROS and mitochondrial ROS at 48 h (Figures 3(e)–3(h)). Hence, these results indicate that mitochondrial dysfunction and distortion caused by HG are blocked by TRAP1.

### 3.4. TRAP1 Protected against Mitochondrial Damage via mPTP Regulation

**Figure 4:**
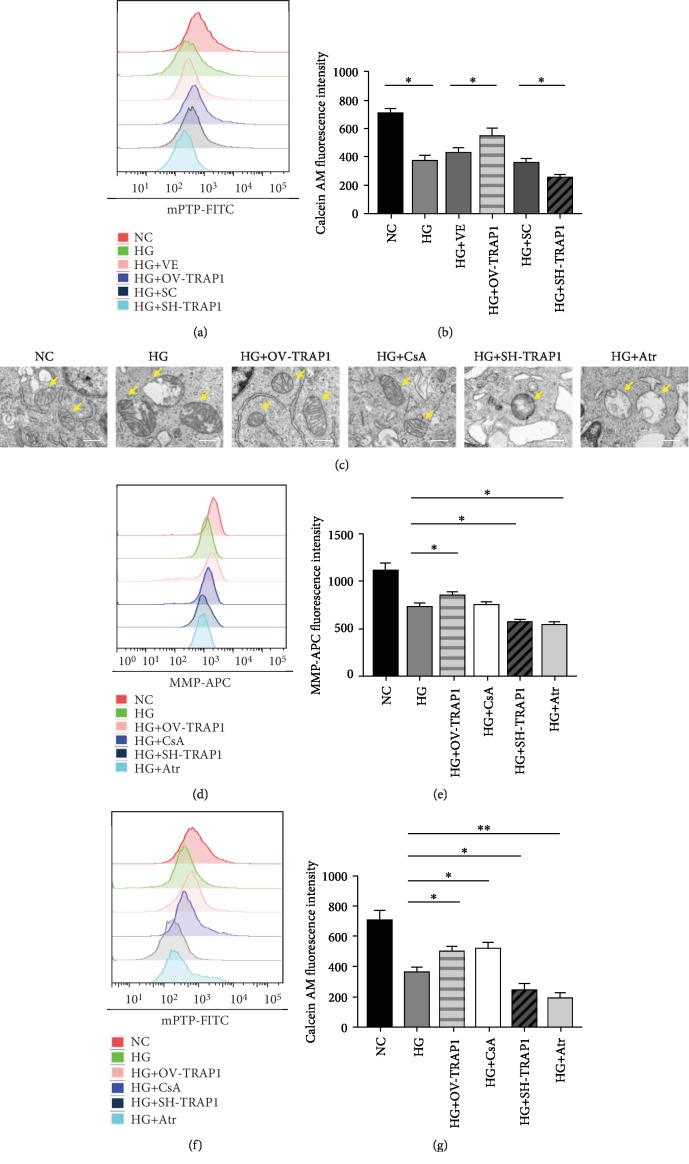
TRAP1 protects against high-glucose-induced mitochondrial dysfunction via mPTP opening regulation. (a, b) Representative graphs and statistical analyses of mPTP by flow cytometry with the fluorescent probe calcein AM labeling. (c) Mitochondrial ultrastructural damage was observed by TEM. Arrows indicate mitochondria; scale bar, 500 nm. (d, e) Typical graphs of mitochondrial membrane potential and statistical analysis determined with tetramethylrhodamine ethyl ester labeling by flow cytometric analysis at 48 h. (f, g) Representative graphs and statistical analysis of mPTP by flow cytometry. mPTP: mitochondrial permeability transition pore. The results are presented as the mean ± SEM; *n* = 3, ^∗^*p* < 0.05, and ^∗∗^*p* < 0.01 for each pair of groups indicated.

Next, we aimed to investigate the mechanism of TRAP1 protection in HG. Opening of the mPTP leads to a cascade of events, including an increase in the permeability of solutes, dissipation of mitochondrial membrane potential, and rupture of the outer membrane with the induction of caspase-3 activity, which eventually initiates apoptosis [18–20]. TRAP1 overexpression suppressed the opening of the mPTP to delay the remaining fluorescence loss from the mitochondria in NRK-52e cells. However, TRAP1 silencing significantly increased the opening of the mPTP (Figures 4(a) and 4(b)). To further determine whether the effect of TRAP1 on mitochondria is mediated by mPTP regulation under HG conditions, we incubated NRK-52e cells with the mPTP opener drug atractyloside (Atr) or a specific inhibitor of mPTP opening cyclosporin A (CsA) [21–23]. Similar to the effect of CsA, the cells transfected with the OV lentivirus showed decreased mitochondrial swelling, as observed by TEM (Figure 4(c)) and mPTP opening inhibition (Figures 4(f) and 4(g)). In contrast, cells transfected with shRNA or the mPTP opener Atr had increased mitochondrial morphological damage and reduced function (Figures 4(c)–4(g)). These results indicated that the effects of TRAP1 overexpression and silencing are similar to those of the mPTP opening regulator, which means that the protective effect of TRAP1 may be exerted via the regulation of mPTP opening.

**Figure 5:**
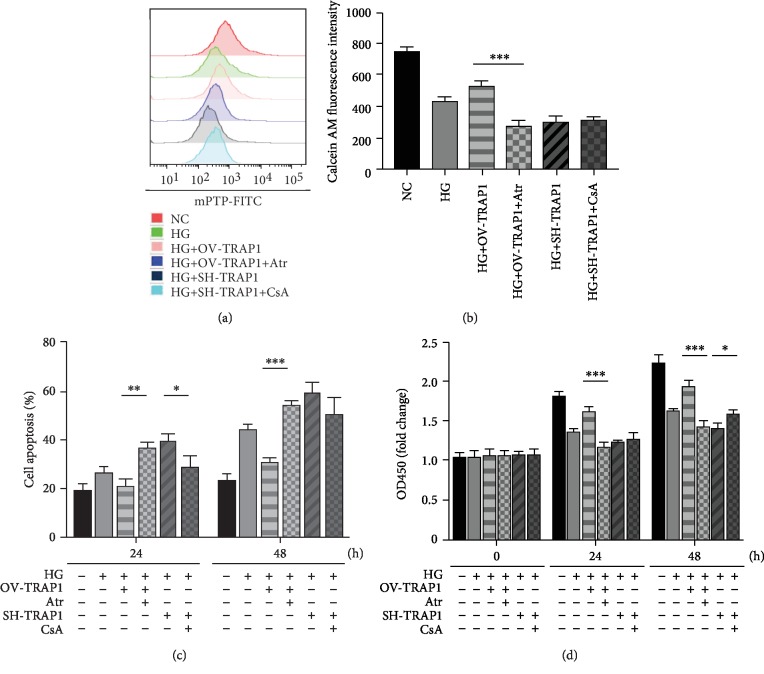
The effects of TRAP1 can be reversed by the mPTP opening regulator. (a, b) Representative graphs and statistical analysis of mPTP by flow cytometry. (c) Statistical analysis of apoptosis rates detected by flow cytometry after APC/7AAD dual staining. (d) The viability of NRK-52e cells was measured using the CCK-8 assay. The results are presented as the mean ± SEM; *n* = 3, ^∗^*p* < 0.05, ^∗∗^*p* < 0.01, and ^∗∗∗^*p* < 0.001 for each pair of groups indicated.

To study the potential target of TRAP1 in DKD and the underlying mechanisms, we tested whether the effect of TRAP1 could be reversed. The mPTP opener Atr and mPTP inhibitor CsA were added to the medium. When Atr was added to the TRAP1-overexpressing cell culture medium, the mPTP opening inhibition, antiapoptosis, and cell viability increase effects were abolished (Figures 5(a)–5(d)). Representative photos of the apoptosis detection results are shown in the supplementary material (ESM Fig. [Supplementary-material supplementary-material-1]). However, treating TRAP1-knockdown cells with CsA did not significantly delay mPTP opening (Figures 5(a) and 5(b)). The knockdown of TRAP1 sensitized NRK-52e cells causing them to undergo apoptosis, and this effect was also antagonized by simultaneous CsA treatment only at 24 h (Figure 5(c)). Likewise, CCK-8 release analysis showed that CsA markedly reversed the TRAP1-knockdown effect on cell viability at 48 h (Figure 5(d)). These results demonstrate that TRAP1 prevents mitochondrial damage via mPTP regulation and that the aggravating damage caused by TRAP1 knockdown can be partially reversed by treatment with the mPTP opening inhibitor CsA, indicating a recoverable target for DKD treatment.

### 3.5. TRAP1 Overexpression Prevents Renal Dysfunction and Histopathological Changes in Diabetic Rats

**Figure 6:**
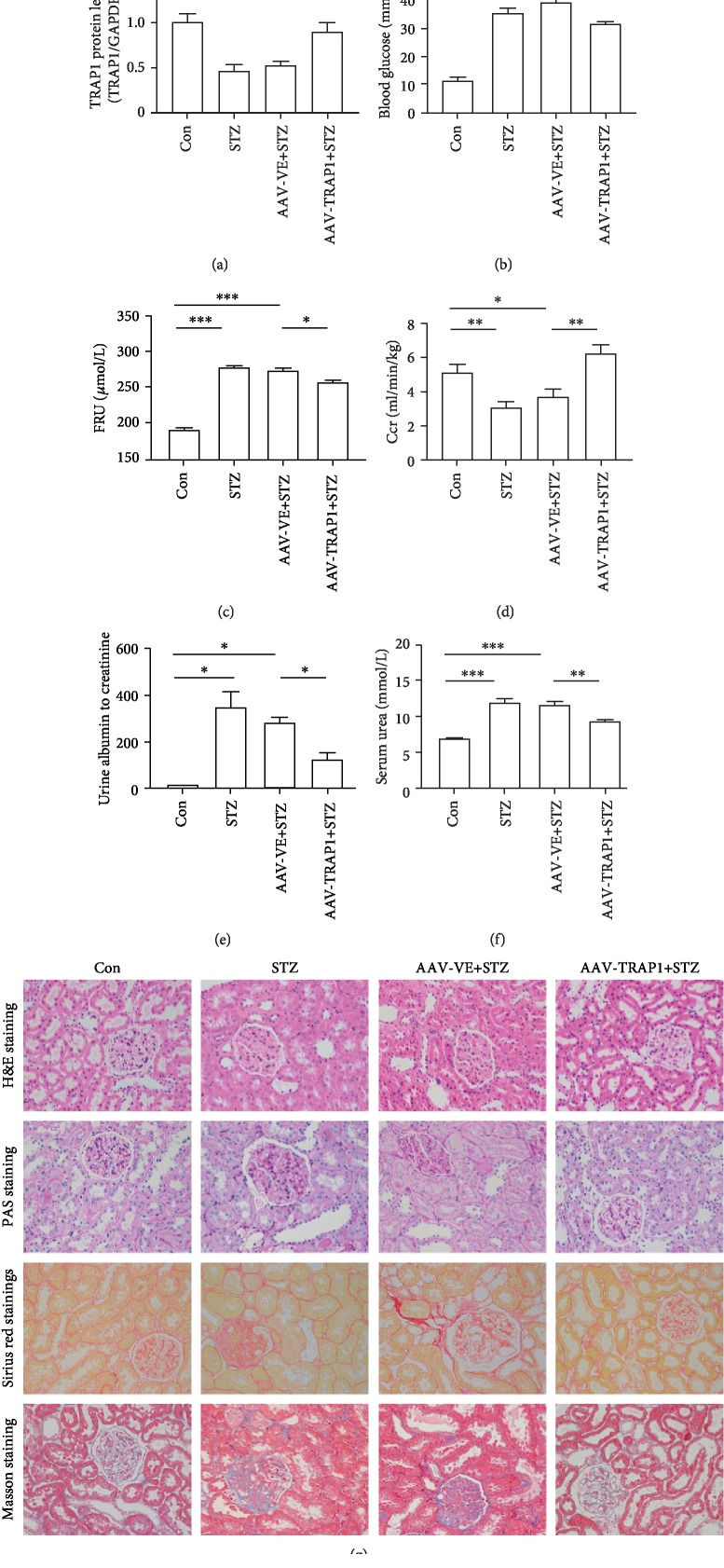
Effect of TRAP1 overexpression on biochemical parameters and histopathology in vivo. Rats were treated with an injection of STZ to induce diabetes and AAV 2/9 to overexpress TRAP1. Rats transfected with empty-GFP vectors (AAV-VE) are shown for comparison. Blood and kidney tissues were collected after 12 weeks. (a) Western blot graphs and densitometric analyses of TRAP1 expression in kidneys. GAPDH was used as a control. (b–f) Blood glucose (b), serum FRU (c), serum Ccr (d), urine albumin to creatinine (e), and serum urea (f) levels were analyzed. (g) Representative histology of the renal cortex and outer medulla: H&E staining, PAS for glycogen, Sirius red staining for the detection of fibrosis, and Masson's trichrome staining for connective tissue; scale bar: 50 *μ*m. The results are presented as the mean ± SEM; *n* = 4‐6, ^∗^*p* < 0.05, ^∗∗^*p* < 0.01, and ^∗∗∗^*p* < 0.001 for each pair of groups indicated.

To test the role of TRAP1 in diabetes-associated kidney damage, rats were treated with STZ and then fed for 12 weeks to develop DKD. Simultaneously, in this model, we overexpressed TRAP1 by injecting AAV2/9 vectors expressing TRAP1 labeled with EGFP. Control animals were injected with AAV vectors encoding EGFP alone. Observation of the GFP staining showed viral expression in renal tubular epithelial cells (ESM Fig. [Supplementary-material supplementary-material-1]). The level of TRAP1 in diabetic rat kidneys was lower than that in nondiabetic control rat kidneys, consistent with the results in cell lines. Increased TRAP1 expression was detected in AAV-TRAP1-injected rats compared to negative control-injected rats (Figure 6(a)), indicating the successful transfection of TRAP1 in diabetic rats. The biochemical indexes and histological examination confirmed the characteristics of renal tubule damage in diabetic rats. The renal indexes (RI), urine output, total cholesterol (TC), total triglyceride (TG), low-density lipoprotein (LDL), blood glucose, fructosamine (FRU), urinary albumin to creatinine, and serum urea were significantly increased in diabetic rats, whereas the body weight, high-density lipoprotein (HDL), and creatinine clearance rate (Ccr) were lower than those in the control rats (Figures 6(b)–6(f), ESM [Supplementary-material supplementary-material-1]). Compared to the empty vector-expressing diabetic rats, TRAP1-overexpressing diabetic rats had an increased Ccr and decreased levels of blood glucose, FRU, urine albumin to creatinine, and serum urea at the end of the 12^th^ week (Figures 6(b)–6(f)). However, the upregulation of TRAP1 expression had no significant effect on the body weight, RI, urine output, TC, TG, LDL, or HDL (ESM [Supplementary-material supplementary-material-1]). These results indicate that TRAP1 overexpression decreases renal injury in STZ-induced diabetic rats.

The evaluation of the histopathological changes of rat kidney was used to examine renal injury amelioration by the upregulation of TRAP1. Histological analysis showed that diabetes-induced glycogen accumulation and structural damage were prevented by TRAP1 overproduction. The histological analysis by hematoxylin-eosin (H&E) staining and periodic acid-Schiff (PAS) staining revealed that diabetes led to proximal tubular cell atrophy, tubular vacuolization, increased tubulointerstitial fibrosis, tubular epithelial apoptosis and necrosis, thickening of the tubular basement membrane, and glomerular sclerosis. Similarly, Sirius red staining and Masson's trichrome staining revealed that collagen expression and fibrosis were enhanced in diabetic rats, whereas these abnormalities were attenuated in TRAP1-overexpressing rats (Figure 6(g)). In short, these findings confirm the renoprotective effects of TRAP1 overexpression in diabetic rats.

### 3.6. TRAP1 Attenuates Apoptosis and Mitochondrial Damage in Diabetic Rats

**Figure 7:**
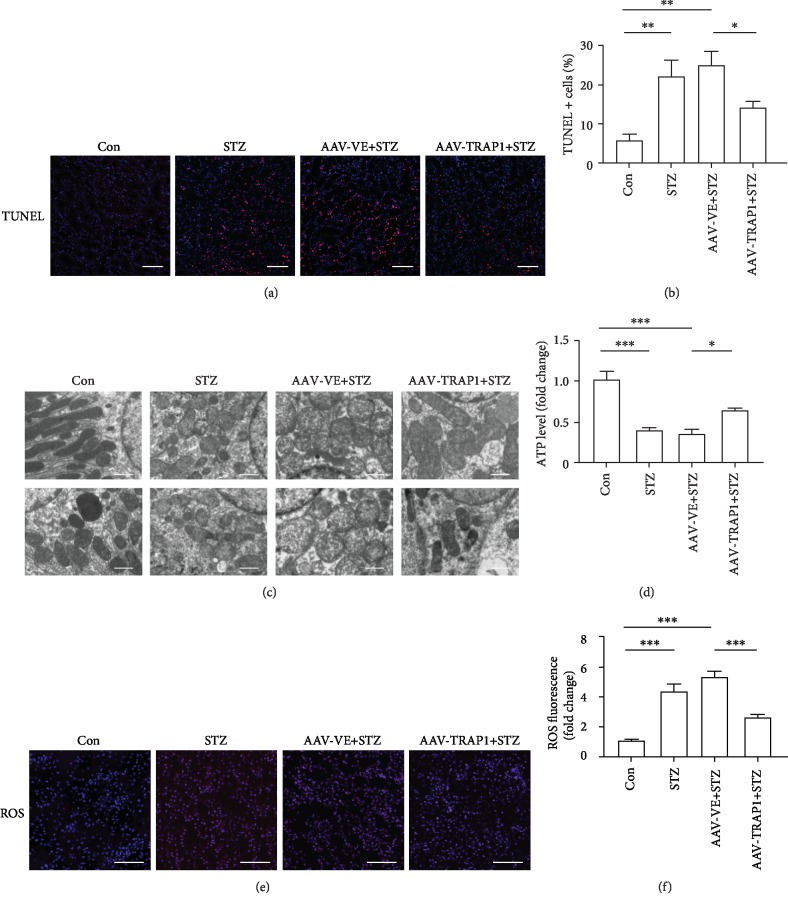
Effects of TRAP1 on diabetes-induced apoptosis and mitochondrial damage in rat kidney. (a) Representative images of (TUNEL) staining (red: TUNEL; blue: DAPI); scale bar, 100 *μ*m. (b) Quantification of TUNEL-positive cells in fields. (c) Mitochondria of tubular epithelial cells viewed by TEM; scale bar, 1 *μ*m. (d) ATP levels measured by firefly luciferase detection. (e, f) Representative photomicrographs and quantification of ROS staining in rat renal tubules; scale bar, 100 *μ*m. The results are presented as the mean ± SEM; *n* = 4‐6, ^∗^*p* < 0.05, ^∗∗^*p* < 0.01, and ^∗∗∗^*p* < 0.001 for each pair of groups indicated.

In our study, TRAP1 expression upregulation markedly attenuated apoptosis in the renal tubules of diabetic rats (Figures 7(a) and 7(b)). In addition, electron microscopy data showed that the mitochondria of proximal tubular cells were injured in diabetic rat renal cortexes. The mitochondria presented with abnormal size and arrangements, including swelling and vacuoles. TRAP1 expression upregulation improved the recovery of injured mitochondria to some degree (Figure 7(c)). Furthermore, ATP levels in the renal cortex were significantly reduced in the diabetes group. TRAP1-overexpressing rats had increased ATP levels compared to control vector-expressing rats (Figure 7(d)). For ROS level staining, TRAP1 overexpression decreased the ROS generation in the kidney tissues of diabetic rats (Figures 7(e) and 7(f)). These findings indicate that the inhibitory effects of TRAP1 overexpression on tubular cell apoptosis and mitochondria dysfunction may account for the improvement of renal function. The upregulation of TRAP1 expression can ameliorate apoptosis and mitochondrial damage in diabetic rats, consistent with the results in vitro experiments.

## 4. Discussion

DKD remains a substantial clinical issue and has led to an increased economic burden worldwide. In this study, we found that TRAP1 improved renal injury in diabetic rats and reduced renal tubular epithelial cell apoptosis by maintaining mitochondrial structure and function, which may be associated with the regulation of mPTP opening.

Abnormalities in mitochondrial function play a central role in the pathogenesis of complications of diabetes [2, 7]. Renal tubular epithelial cells are sensitive to environmental injury and require much ATP produced by mitochondria. In our study, mitochondria were compromised in renal tubular epithelial cells exposed to HG, with concomitant cellular injury and apoptosis. Apoptotic stimuli can damage mitochondria [24, 25]. Mitochondrial dysfunction can lead to apoptosis, which may be the predominant mechanism of epithelial cell death in DKD [4]. Thus, there is an urgent need to protect mitochondria in diabetes. TRAP1, a mitochondrial molecular chaperone, has been confirmed to have complex and numerous functions [26, 27]. It has also been proven to play important roles in tubular epithelial cell mitochondrial injury induced by unilateral ureteral obstruction [28]. However, whether TRAP1 is involved in the regulation of renal tubule damage in DKD remains undefined.

First, this study showed that TRAP1 significantly ameliorates diabetes-induced renal injury, and this protection may be correlated with its antiapoptotic effects on renal tubular cells. Treatment with HG decreased the level of TRAP1, which is consistent with the results in diabetic rats. HG has been implicated as a mediator of apoptosis [29, 30]. TRAP1 overexpression increased cell viability, decreased apoptosis, and downregulated cleaved caspase-3 activity, which is a major mediator of proximal tubule apoptosis [31]. In vivo, the proximal tubule undergoes a range of structural changes, including interstitial fibrosis, tubular atrophy, and peritubular capillary rarefaction, which are closely related to kidney function decline [32]. TRAP1 overexpression attenuated the damage in diabetic rats as assessed by both biochemical indexes and histological staining. Similar to the results of the observations in vitro, apoptotic cell numbers in kidneys were decreased in TRAP1-overexpressing diabetic rats. However, we were unable to confirm which types of cell and the specific mechanism responsible for the effect of TRAP1 on rat kidney tissues in vivo study. As a conserved protein, TRAP1 may also affect other cell types in kidney tissues, which needs to be confirmed in future studies.

Second, the effects of TRAP1 on HG-induced mitochondrial injury were investigated in our study and may be the mechanism underlying its antiapoptotic effects. TEM was used for mitochondrial morphology evaluation in our study [33]. The electron microscopy studies indicated that mitochondria are enlarged in diabetic kidney tubules in humans [34]. A reduced density of mitochondria and mitochondrial swelling represent indicators of mitochondrial dysfunction [35]. In addition, MMP and ATP are sensitive indicators of mitochondrial function. Our results indicate that TRAP1 protects against mitochondrial morphological and functional damage under HG conditions. Mitochondria are considered to be a vital source of oxidative stress. The excess production of ROS results in oxidative damage to mitochondrial proteins and DNA [36] and ultimately renal damage. The generation of ROS exceeding local antioxidant capacity has been reported to be a mitochondrial dysfunction biomarker in DKD [37–39]. Exposure to oxidative stress inducers upregulate mitochondrial ROS production. In previous studies, TRAP1 expression and ROS levels were shown to be inversely correlated, which may be the consequence of mitochondrial respiration regulation [40–42]. Indeed, we found that TRAP1 overexpression reduced ROS production in mitochondria. Similar to that in vitro, mitochondrial dysfunction was minimized by TRAP1 expression upregulation in diabetic rats, which further proves the role TRAP1 plays in DKD.

Third, the specific mechanisms by which TRAP1 protects mitochondria were studied. The mPTP is an elusive nonselective pore for water and small solutes that is impermeable under normal conditions but opens suddenly when exposed to stress [43]. The modulation of the mPTP has been implicated as a therapeutic approach in multiple diseases [20, 44]. It has been reported to be regulated by TRAP1 in hypoxic injury [10]. Opening of the mPTP is a key prerequisite for the induction of mitochondrial-mediated apoptosis and is of critical importance during HG injury [45]. According to our results, TRAP1 inhibited mPTP opening, which may be the mechanism by which TRAP1 protects mitochondria against HG-induced stress. TRAP1 overexpression protected against mitochondrial damage, similar with the selective mPTP opening inhibitor CsA. The aggravation of mitochondrial injury was similar in TRAP1 shRNA- and mPTP opener Atr-treated cells. Above all, TRAP1 can block the process of mitochondrial permeability transition (mPT) and protect against the subsequent cascade of events, which include an increase in solute permeability, swelling of the mitochondrial matrix, dissipation of the transmembrane potential, and rupture of the outer membrane with the induction of caspase-3 activity, which eventually initiates apoptosis [20]. Next, to confirm whether the effects of negating apoptosis can be altered by regulating mPTP opening, we incubated treated cells with Atr or CsA. Our results showed that CsA ameliorated TRAP1 knockdown-induced apoptosis, whereas Atr reversed the TRAP1-induced antiapoptotic effects, which indicates that TRAP1 is a recoverable target for DKD treatment.

## 5. Conclusion

In summary, our study provides compelling evidence that TRAP1 could improve renal function, ameliorate histopathological changes, decrease apoptosis, restore mitochondrial damage, and buffer oxidative stress in diabetic rat kidneys, which is attributable to the role TRAP1 plays in blocking mPTP opening and maintaining mitochondrial structure and function. The findings from the current study establish a novel role of TRAP1 in tubular epithelial protection through the mitochondrial machinery and may have implications for DKD therapy.

## Data Availability

The data used to support the findings of this study are included within the article.

## Abbreviations

AAV: Adeno-associated virus
Atr: Atractyloside
CsA: Cyclosporin A
Ccr: Creatinine clearance rate
DKD: Diabetic kidney disease
ESRD: End-stage renal disease
GFP: Green fluorescent protein
HDL: High-density lipoprotein
H&E: Hematoxylin and eosin
HG: High glucose
HSP 90: Heat-shock protein 90
LDL: Low-density lipoprotein
MMP: Mitochondrial membrane potential
mPT: Mitochondrial permeability transition
mPTP: Mitochondrial permeability transition pore
PAS: Periodic acid- schiff
RI: Renal index
qPCR: Quantitative PCR
ROS: Reactive oxygen species
STZ: Streptozotocin
TC: Total cholesterol
TEM: Transmission electron microscopy
TG: Total triglyceride
TRAP1: Tumor necrosis factor receptor-associated protein 1
TUNEL: Terminal deoxynucleotidyl transferase-mediated dUTP nick-end labeling.

## Supplementary Materials

Supplementary Materials ESM Table S1: body weight, KI, and biochemical indicators from the normal control (Con), STZ-induced diabetic groups (STZ), and rats infected with AAV-VE or AAV-TRAP1 at 12 weeks. ESM Figure S1: effects of high glucose on mitochondria membrane potential in NRK-52e cells at 48 h. Typical fluorescence photomicrographs of mitochondrial membrane potential after JC-1 staining. Scale bar = 250 *μ*m. ESM Figure S2: effects of TRAP1 on apoptosis exposed to high glucose in NRK-52e cells. TRAP1 overexpression and knockdown cells were cultured under high-glucose conditions. Typical graphs of apoptosis were detected by flow cytometry after APC/7AAD dual staining. ESM Figure S3: effects of mPTP opening regulator on apoptosis of TRAP1 overexpression and knockdown cells. Typical graphs of apoptosis were detected by flow cytometry after APC/7AAD staining. ESM Figure S4: the efficiency of AAV 2/9 infection in vivo. Typical fluorescence photomicrographs of GFP in rat renal tubular epithelial cells. Scale bar = 250 *μ*m.

## References

[1] Saran R., Robinson B., Abbott K. C. (2019). US renal data system 2018 annual data report: epidemiology of kidney disease in the United States. *American Journal of Kidney Diseases*.

[2] Forbes J. M., Thorburn D. R. (2018). Mitochondrial dysfunction in diabetic kidney disease. *Nature Reviews: Nephrology*.

[3] Gilbert R. E. (2017). Proximal tubulopathy: prime mover and key therapeutic target in diabetic kidney disease. *Diabetes*.

[4] Kelly D. J., Cox A. J., Tolcos M., Cooper M. E., Wilkinson-Berka J. L., Gilbert R. E. (2002). Attenuation of tubular apoptosis by blockade of the renin-angiotensin system in diabetic Ren-2 rats. *Kidney International*.

[5] Kumar D., Robertson S., Burns K. D. (2004). Evidence of apoptosis in human diabetic kidney. *Molecular and Cellular Biochemistry*.

[6] Jeong S. Y., Seol D. W. (2008). The role of mitochondria in apoptosis. *BMB Reports*.

[7] Bhargava P., Schnellmann R. G. (2017). Mitochondrial energetics in the kidney. *Nature Reviews: Nephrology*.

[8] Altieri D. C., Stein G. S., Lian J. B., Languino L. R. (2012). TRAP-1, the mitochondrial Hsp90. *Biochimica et Biophysica Acta*.

[9] Amoroso M. R., Matassa D. S., Sisinni L., Lettini G., Landriscina M., Esposito F. (2014). TRAP1 revisited: novel localizations and functions of a ‘next-generation’ biomarker (review). *International Journal of Oncology*.

[10] Xiang F., Huang Y. S., Shi X. H., Zhang Q. (2010). Mitochondrial chaperone tumour necrosis factor receptor-associated protein 1 protects cardiomyocytes from hypoxic injury by regulating mitochondrial permeability transition pore opening. *The FEBS Journal*.

[11] Voloboueva L. A., Duan M., Ouyang Y., Emery J. F., Stoy C., Giffard R. G. (2008). Overexpression of mitochondrial Hsp70/Hsp75 protects astrocytes against ischemic injury in vitro. *Journal of Cerebral Blood Flow and Metabolism*.

[12] Stacchiotti A., Ricci F., Rezzani R. (2006). Tubular stress proteins and nitric oxide synthase expression in rat kidney exposed to mercuric chloride and melatonin. *The Journal of Histochemistry and Cytochemistry*.

[13] Pak M. G., Koh H. J., Roh M. S. (2017). Clinicopathologic significance of TRAP1 expression in colorectal cancer: a large scale study of human colorectal adenocarcinoma tissues. *Diagnostic Pathology*.

[14] Lisanti S., Garlick D. S., Bryant K. G. (2016). Transgenic expression of the mitochondrial chaperone TNFR-associated protein 1 (TRAP1) accelerates prostate cancer development. *The Journal of Biological Chemistry*.

[15] Im C. N., Seo J. S. (2014). Overexpression of tumor necrosis factor receptor-associated protein 1 (TRAP1), leads to mitochondrial aberrations in mouse fibroblast NIH/3T3 cells. *BMB Reports*.

[16] Matassa D. S., Amoroso M. R., Lu H. (2016). Oxidative metabolism drives inflammation-induced platinum resistance in human ovarian cancer. *Cell Death and Differentiation*.

[17] Zhou B., Li Q., Wang J., Chen P., Jiang S. (2019). Ellagic acid attenuates streptozocin induced diabetic nephropathy via the regulation of oxidative stress and inflammatory signaling. *Food and Chemical Toxicology*.

[18] Zorov D. B., Juhaszova M., Yaniv Y., Nuss H. B., Wang S., Sollott S. J. (2009). Regulation and pharmacology of the mitochondrial permeability transition pore. *Cardiovascular Research*.

[19] Baumgartner H. K., Gerasimenko J. V., Thorne C. (2009). Calcium elevation in mitochondria is the main Ca2+ requirement for mitochondrial permeability transition pore (mPTP) opening. *The Journal of Biological Chemistry*.

[20] Morciano G., Giorgi C., Bonora M. (2015). Molecular identity of the mitochondrial permeability transition pore and its role in ischemia-reperfusion injury. *Journal of Molecular and Cellular Cardiology*.

[21] Mishra J., Davani A. J., Natarajan G. K., Kwok W. M., Stowe D. F., Camara A. K. S. (2019). Cyclosporin a increases mitochondrial buffering of calcium: an additional mechanism in delaying mitochondrial permeability transition pore opening. *Cell*.

[22] Ma L., Niu W., Yang S. (2018). Inhibition of mitochondrial permeability transition pore opening contributes to cannabinoid type 1 receptor agonist ACEA-induced neuroprotection. *Neuropharmacology*.

[23] Fan Y. Y., Shen Z., He P. (2014). A novel neuroprotective strategy for ischemic stroke: transient mild acidosis treatment by CO2 inhalation at reperfusion. *Journal of Cerebral Blood Flow and Metabolism*.

[24] Green D. R., Kroemer G. (2004). The pathophysiology of mitochondrial cell death. *Science*.

[25] Ly J. D., Grubb D. R., Lawen A. (2003). The mitochondrial membrane potential (deltapsi(m)) in apoptosis; an update. *Apoptosis*.

[26] Matassa D. S., Agliarulo I., Avolio R., Landriscina M., Esposito F. (2018). TRAP1 regulation of cancer metabolism: dual role as oncogene or tumor suppressor. *Genes*.

[27] Fitzgerald J. C., Zimprich A., Carvajal Berrio D. A. (2017). Metformin reverses TRAP1 mutation-associated alterations in mitochondrial function in Parkinson's disease. *Brain*.

[28] Chen J. F., Wu Q. S., Xie Y. X. (2017). TRAP1 ameliorates renal tubulointerstitial fibrosis in mice with unilateral ureteral obstruction by protecting renal tubular epithelial cell mitochondria. *The FASEB Journal*.

[29] Ortiz A., Ziyadeh F. N., Neilson E. G. (1997). Expression of apoptosis-regulatory genes in renal proximal tubular epithelial cells exposed to high ambient glucose and in diabetic kidneys. *Journal of Investigative Medicine*.

[30] Lorenzi M., Montisano D. F., Toledo S., Barrieux A. (1986). High glucose induces DNA damage in cultured human endothelial cells. *The Journal of Clinical Investigation*.

[31] Cryns V., Yuan J. (1998). Proteases to die for. *Genes & Development*.

[32] Gilbert R. E., Cooper M. E. (1999). The tubulointerstitium in progressive diabetic kidney disease: more than an aftermath of glomerular injury?. *Kidney International*.

[33] Li X., Lao Y., Zhang H. (2015). The natural compound Guttiferone F sensitizes prostate cancer to starvation induced apoptosis via calcium and JNK elevation. *BMC Cancer*.

[34] Takebayashi S., Kaneda K. (1991). Mitochondrial derangement: possible initiator of microalbuminuria in NIDDM. *The Journal of Diabetic Complications*.

[35] Hall A. M., Rhodes G. J., Sandoval R. M., Corridon P. R., Molitoris B. A. (2013). In vivo multiphoton imaging of mitochondrial structure and function during acute kidney injury. *Kidney International*.

[36] Forbes J. M., Coughlan M. T., Cooper M. E. (2008). Oxidative stress as a major culprit in kidney disease in diabetes. *Diabetes*.

[37] Coughlan M. T., Nguyen T. V., Penfold S. A. (2016). Mapping time-course mitochondrial adaptations in the kidney in experimental diabetes. *Clinical Science*.

[38] Tan A. L., Sourris K. C., Harcourt B. E. (2010). Disparate effects on renal and oxidative parameters following RAGE deletion, AGE accumulation inhibition, or dietary AGE control in experimental diabetic nephropathy. *American Journal of Physiology: Renal Physiology*.

[39] Dugan L. L., You Y. H., Ali S. S. (2013). AMPK dysregulation promotes diabetes-related reduction of superoxide and mitochondrial function. *The Journal of Clinical Investigation*.

[40] Yoshida S., Tsutsumi S., Muhlebach G. (2013). Molecular chaperone TRAP1 regulates a metabolic switch between mitochondrial respiration and aerobic glycolysis. *Proceedings of the National Academy of Sciences of the United States of America*.

[41] Hua G., Zhang Q., Fan Z. (2007). Heat shock protein 75 (TRAP1) antagonizes reactive oxygen species generation and protects cells from granzyme M-mediated apoptosis. *The Journal of Biological Chemistry*.

[42] Masuda Y., Shima G., Aiuchi T. (2004). Involvement of tumor necrosis factor receptor-associated protein 1 (TRAP1) in apoptosis induced by beta-hydroxyisovalerylshikonin. *The Journal of Biological Chemistry*.

[43] Seidlmayer L. K., Juettner V. V., Kettlewell S., Pavlov E. V., Blatter L. A., Dedkova E. N. (2015). Distinct mPTP activation mechanisms in ischaemia-reperfusion: contributions of Ca2+, ROS, pH, and inorganic polyphosphate. *Cardiovascular Research*.

[44] Du H., Guo L., Fang F. (2008). Cyclophilin D deficiency attenuates mitochondrial and neuronal perturbation and ameliorates learning and memory in Alzheimer's disease. *Nature Medicine*.

[45] Deshwal S., Forkink M., Hu C. H. (2018). Monoamine oxidase-dependent endoplasmic reticulum-mitochondria dysfunction and mast cell degranulation lead to adverse cardiac remodeling in diabetes. *Cell Death and Differentiation*.

