# Supplementary material for: Tumor Necrosis Factor Receptor-Associated Protein 1 Protects against Mitochondrial Injury by Preventing High Glucose-Induced mPTP Opening in Diabetes
Source: Oxid Med Cell Longev. 2020 Mar 6;2020:6431517. doi: 10.1155/2020/6431517 (PMC7079224; doi:10.1155/2020/6431517)
Supplement: Supplementary Materials — ESM Table S1: body weight, KI, and biochemical indicators from the normal control (Con), STZ-induced diabetic groups (STZ), and rats infected with AAV-VE or AAV-TRAP1 at 12 weeks. ESM Figure S1: effects of high glucose on mitochondria membrane potential in NRK-52e cells at 48 h. Typical fluorescence photomicrographs of mitochondrial membrane potential after JC-1 staining. Scale bar = 250 μm. ESM Figure S2: effects of TRAP1 on apoptosis exposed to high glucose in NRK-52e cells. TRAP1 overexpression and knockdown cells were cultured under high-glucose conditions. Typical graphs of apoptosis were detected by flow cytometry after APC/7AAD dual staining. ESM Figure S3: effects of mPTP opening regulator on apoptosis of TRAP1 overexpression and knockdown cells. Typical graphs of apoptosis were detected by flow cytometry after APC/7AAD staining. ESM Figure S4: the efficiency of AAV 2/9 infection in vivo. Typical fluorescence photomicrographs of GFP in rat renal tubular epithelial cells. Scale bar = 250 μm. [file 6431517.f1.pdf]

Contents

ESM Table 1

ESM Figure S1

ESM Figure S2

ESM Figure S3

ESM Figure S4

ESM Table S1

Body weight, KI, and biochemical indicators from the normal control (Con), STZ induced diabetic groups (STZ), and rats infected with AAV-VE or AAV-TRAP1 at 12 weeks

Parameters	Con	STZ	STZ+AAV-VE	STZ+AAV-TRAP1
Body weight (g)	484.33±4.22	274.00±22.22 [aaa]	168.67±13.84[aaa]	179.00±14.75[aaa]
RI (g/kg)	3.88±0.06	7.04±0.32[aa]	8.49±0.34[aa]	7.55±0.51[aa]
Urine output (ml/24h)	12±0.84	65.6±16.27	67±11.52[a]	61.4±3.56[aaa]
TC (mmol/L)	1.57±0.02	2.45±0.26[aa]	2.47±0.19[aa]	2.32±0.25[a]
TG (mmol/L)	1.22±0.05	7.13±2.28	8.44±1.42[a]	5.80±0.64[aa]
LDL (mmol/L)	0.33±0.02	0.44±0.03[a]	0.58±0.03[aaa]	0.53±0.04[aaa]
HDL (mmol/L)	1.27±0.02	1.11±0.03[a]	0.86±0.08[a]	1.00±0.02[aaa]

RI (KW/BW) = [left kidney weight (mg)+ right kidney weight (mg)]/(2*body weight)(g) ; TC, total cholesterol; TG, total triglyceride; LDL-C, low density lipoprotein; HDL, high density lipoprotein. Values in the table are presented as means ± SEM (n=5-6). [a] $p < 0.05$, [aa] $p < 0.01$ and [aaa] $p < 0.001$ compared to normal rats respectively.

ESM Fig.S1

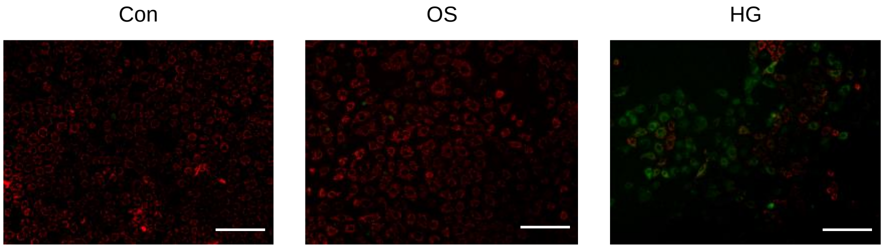

ESM Fig.S1. Effects of high glucose on mitochondria membrane potential in NRK-52e cells at 48h.

Typical fluorescence photomicrographs of mitochondrial membrane potential after JC-1 staining.

Scale bar =250 μm.

ESM Fig.S2

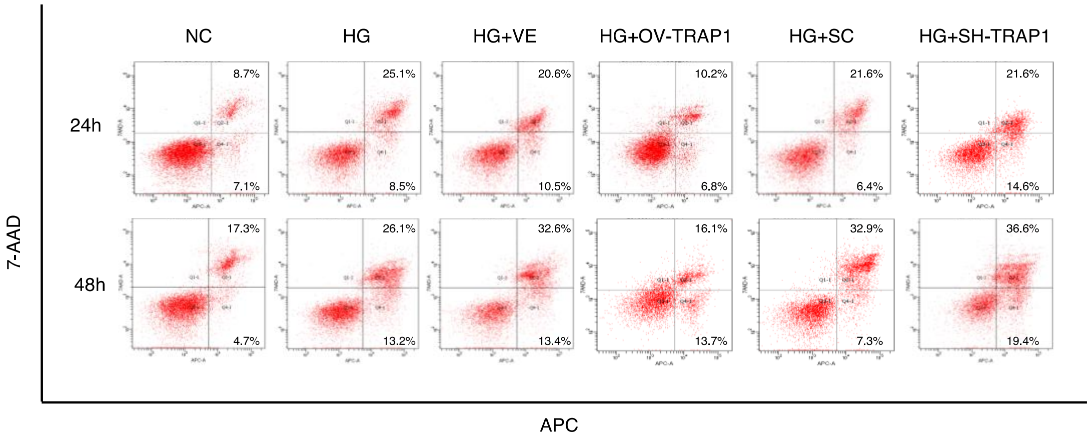

ESM Fig.S2. Effects of TRAP1 on apoptosis exposed to high glucose in NRK-52e cells. TRAP1 overexpression and knockdown cells were cultured under high glucose conditions. Typical graphs of apoptosis were detected by flow cytometry after APC/7AAD dual staining.

ESM Fig.S3

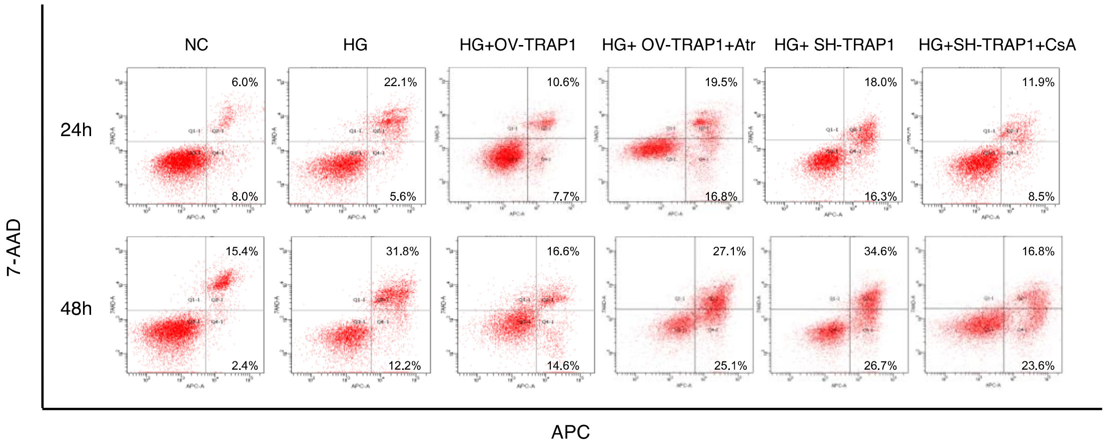

ESM Fig.S3. Effects of mPTP opening regulator on apoptosis of TRAP1 overexpression and knockdown cells. Typical graphs of apoptosis were detected by flow cytometry after APC/7AAD staining.

ESM Fig.S4

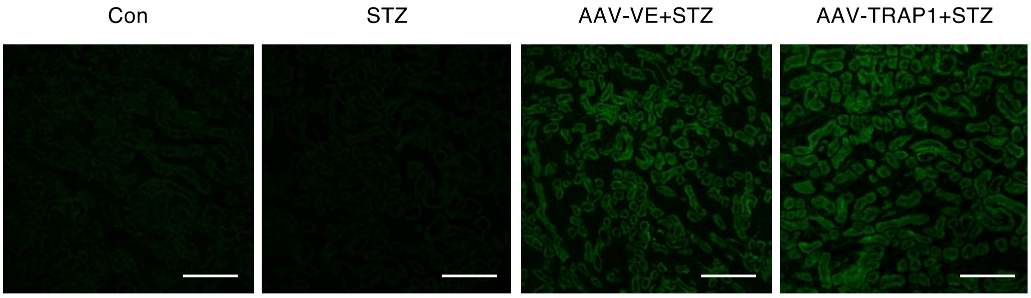

ESM Fig.S4. The efficiency of AAV 2/9 infection in vivo. Typical fluorescence photomicrographs of GFP in rat renal tubular epithelial cells. Scale bar = 250 μm.
